# The Anatomical Distribution of Mechanoreceptors in Mouse Hind Paw Skin and the Influence of Integrin α1β1 on Meissner-Like Corpuscle Density in the Footpads

**DOI:** 10.3389/fnana.2021.628711

**Published:** 2021-03-02

**Authors:** Valerie Wai, Lauren Roberts, Jana Michaud, Leah R. Bent, Andrea L. Clark

**Affiliations:** Department of Human Health and Nutritional Sciences, College of Biological Science, University of Guelph, Guelph, ON, Canada

**Keywords:** Merkel cell, integrin α1β1, mechanoreceptor, hind paw, skin, mouse, footfall, Meissner-like corpuscle

## Abstract

Afferent neurons and their mechanoreceptors provide critical sensory feedback for gait. The anatomical distribution and density of afferents and mechanoreceptors influence sensory feedback, as does mechanoreceptor function. Electrophysiological studies of hind paw skin reveal the different types of afferent responses and their receptive fields, however, the anatomical distribution of mechanoreceptor endings is unknown. Also, the role of integrin α1β1 in mechanoreceptor function is unclear, though it is expressed by keratinocytes in the stratum basale where it is likely involved in a variety of mechanotransduction pathways and ion channel functionalities. For example, it has been shown that integrin α1β1 is necessary for the function of TRPV4 that is highly expressed by afferent units. The purpose of this study, therefore, was to determine and compare the distribution of mechanoreceptors across the hind paw skin and the footfall patterns of *itga1*-null and wild type mice. The *itga1*-null mouse is lacking the integrin α1 subunit, which binds exclusively to the β1 subunit, thus rendering integrin α1β1 nonfunctional while leaving the numerous other pairings of the β1 subunit undisturbed. Intact hind paws were processed, serially sectioned, and stained to visualize mechanoreceptors. Footfall patterns were analyzed as a first step in correlating mechanoreceptor distribution and functionality. Merkel cells and Meissner-like corpuscles were present, however, Ruffini endings and Pacinian corpuscles were not observed. Meissner-like corpuscles were located exclusively in the glabrous skin of the footpads and digit tips, however, Merkel cells were found throughout hairy and glabrous skin. The increased density of Merkel cells and Meissner-like corpuscles in footpads 1 and 3 and Meissner-like corpuscles in footpad 4 suggests their role in anteroposterior balance, while Meissner-like corpuscle concentrations in digits 2 and 5 support their role in mediolateral balance. Finally, a larger density of Meissner-like corpuscles in footpads 3 and 4 in male *itga1*-null mice compared to wild type controls paves the way for future site-specific single fiber *in vivo* recordings to provide insight into the role of integrin α1β1 in tactile mechanotransduction.

## Introduction

Afferent neurons and their mechanoreceptor endings in the glabrous skin of the foot sole are critical to providing the sensory information that is necessary for stance and gait (Strzalkowski et al., 2015, 2018). The anatomical distribution and density of afferents and mechanoreceptors influence sensory feedback, as does mechanoreceptor function. Four classes of low-threshold mechanoreceptors have been described in glabrous skin, including the human foot sole: rapidly adapting type I (RAI) afferents terminating at Meissner’s corpuscles, slowly adapting type I (SAI) afferents terminating at Merkel cells, RAII afferents terminating at Pacinian corpuscles, and SAII afferents terminating at Ruffini endings (Johnson, 2001). SA afferents have a prolonged response that lasts for the duration of the stimulus whereas RA afferents respond briefly at the beginning and end of a stimulus (Rice and Albrecht, 2008). RA afferents are very limited in spatial resolution but respond to dynamic skin deformation, stretch, and low (Meissner’s corpuscles) or high (Pacinian corpuscles) frequency vibration. Meissner’s corpuscles provide critical feedback for grip control, responding to slip between the skin and an object (Johnson, 2001). In contrast, SA afferents resolve spatial details well. Merkel cells can discriminate points, edges, and textures and respond to sustained indentation in proportion to the indentation depth. Ruffini endings however are less sensitive to skin indentation than Merkel cells, but more sensitive to skin stretch perceiving the direction of skin stretch caused by an object or surface, and the position of the digits relative to one another (Johnson, 2001).

Microneurography studies have identified larger afferent proportions innervating the anterolateral foot sole of humans that are postulated to provide mechanosensitive feedback to maintain balance during gait as foot contact moves from the heel to the lateral arch then finally to the digits (Strzalkowski et al., 2018). Parallel electrophysiological studies in specialized *in vivo* and *ex vivo* preparations of the mouse hind paw have identified receptive fields of RA and SA mechanoreceptors in the digits, footpads, and plantar glabrous skin, with a larger proportion of RA responses (71%) compared to SA responses (29%) in all three regions (Cain et al., 2001). Also, neural recordings are reported from RA and SA mechanoreceptors in both hairy and glabrous skin, with equal proportions of both SAI and SAII responses (Walcher et al., 2018). While microneurography and electrophysiology reveal the proportions of different types of afferent responses, the distribution of their receptive fields, and the afferent innervation density in the foot sole or hind paw, inferences are made about mechanoreceptor end-organ distribution in the skin (Strzalkowski et al., 2018). Recordings of a single afferent may not correspond to mechanotransduction from a single mechanoreceptor ending, rather from the neural sum of signals received from multiple branches of a neuron each presumably ending in a mechanoreceptor (Parker and Newsome, 1998; Neubarth et al., 2020). Furthermore, some mechanoreceptors may be innervated by more than one afferent (Vega et al., 2012; Walcher et al., 2018). Thus the exact distribution and density of mechanoreceptor endings in the foot sole and hind paw skin remains unknown and requires direct visualization using histological methods.

In addition to the anatomical distribution and density of afferents and mechanoreceptors, the proper function of the mechanoreceptors themselves is necessary to provide the sensory information for stance and gait. Notwithstanding differences in gait and anatomy between mice and humans, the function of the mechanoreceptors themselves is conserved across mammalian species (Iggo and Andres, 1982). Thus, genetically modified mice can help probe the mechanisms of mechanoreceptor function. The *itga1*-null mouse is lacking the integrin α1 subunit, which binds exclusively to the β1 subunit, thus rendering integrin α1β1 nonfunctional while leaving the numerous other pairings of the β1 subunit undisturbed (Gardner et al., 1996). While the skin sensory phenotype of the *itga1*-null mouse is unknown, keratinocytes express many integrins, including integrin α1β1, together with Merkel cells in the stratum basale. Furthermore, β1 integrins colocalize with Meissner-like corpuscles in the dermal papillae just deep to the stratum basale (Watt and Jones, 1993). Integrins are likely involved in the transduction of forces to the lamellae of the Meissner-like corpuscle *via* collagen fibers, however, their role in Merkel cell function is unknown (Vega et al., 2009; Piccinin et al., 2020). Merkel cells, Meissner-like corpuscles and their associated afferent nerves in the glabrous skin of mouse hind paws are associated with a variety of mechanosensitive ion channels. One ion channel that is highly expressed in these sensory organs is TRPV4 (Suzuki et al., 2003b). Disruption of *trpv4* in mice leads to insensitivity to pressure sensation in the tail (Suzuki et al., 2003a) and interestingly, TRPV4 in *itga1*-null mice is not functional (Jablonski et al., 2014).

The purpose of this study, therefore, was to determine and compare the distribution of mechanoreceptors across the hind paw skin of *itga1*-null and wild type mice. Also, footfall patterns during gait were analyzed as a first step in correlating mechanoreceptor distribution and functionality. We hypothesized that integrin α1β1 increases the extracellular matrix-ion channel connection and thus mechanoreceptor sensitivity. Therefore we expected hind paws from *itga1*-null mice to have more mechanoreceptors compared to wild type mice to compensate for the deficit in mechanoreceptor sensitivity while maintaining typical footfall patterning during gait.

## Materials and Methods

### Ethics and Mice

**Figure 1:**
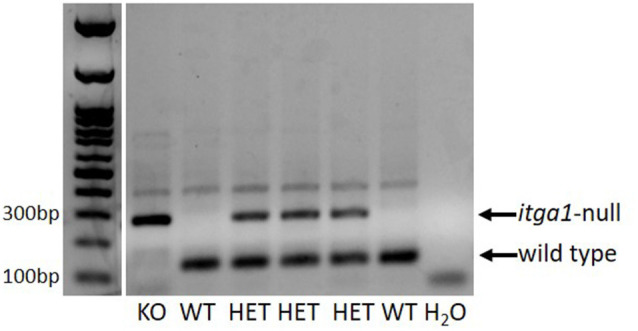
An example PCR gel verifying the genotype of a litter of pups. DNA extracted from identification ear notch samples. In this litter, there was one *itga1-*null (KO), two wild types (WT), and three heterozygous (HET) pups. A blank lane is shown as a negative control (H_2_O).

All animal procedures were approved by the Animal Care Committee of the University of Guelph (AUP#3960). *Itga1*-null and wild type BALB/c mice, generated from pure BALB/c *itga1*-heterozygote breeder pairs established at the University of Guelph, were crossed among themselves to produce the mice for this study. Genotype was determined through a multiplex polymerase chain reaction using DNA extracted from identification ear notches (Figure 1; Gardner et al., 1996). Critically, deletion of the α1 subunit in mice results in the absence of integrin α1β1 due to its exclusive partnership with the β1 subunit but does not influence the expression of the α2, α3, or β1 subunits at the protein level (Gardner et al., 1996; Pozzi et al., 1998). For histological and immunohistochemical assays, eight BALB/c wild type (age = 19 ± 1 week, mass = 26 ± 2.1 g; mean ± SE) and eight *itga1*-null (age = 20 ± 0 weeks, mass = 29 ± 1.6 g; mean ± SE) mice (four of each sex) were anesthetized with isoflurane and euthanized by cardiac puncture followed by cervical dislocation.

### Histology

**Figure 2:**
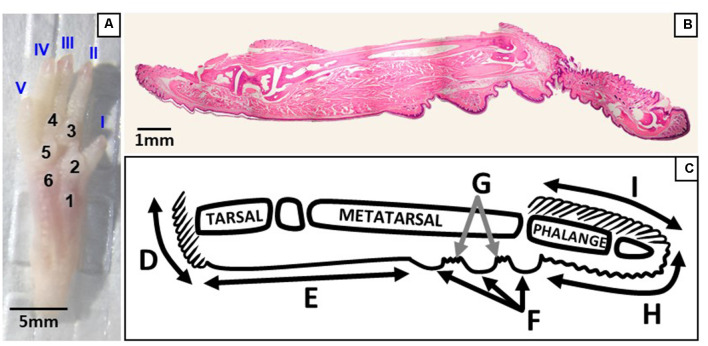
Regions of the plantar and sagittal intact hind paw. **(A)** Photograph of the plantar surface of an intact mouse hind paw showing digits I–V and footpads 1–6. **(B)** Light microscopy image of a whole hind paw in the sagittal plane stained with hematoxylin and eosin. **(C)** Schematic showing the regions of the sagittal view of the hind paw; D, heel hair follicles; E, glabrous plantar metatarsal skin; F, glabrous footpad skin; G, ridged glabrous plantar skin/plantar hair follicles; H, glabrous plantar digit skin; and I, digit hair follicles.

Intact hind paws were prepared as previously described (Wai et al., 2020). Briefly, right hind paws were coated with the chemical depilator Nair^TM^ then rinsed thoroughly with water. Under a dissection microscope (Leica M60, Wetzlar, Germany), the intact hind paw was isolated from the mouse by a transverse cut at the articulation of the tibia and the intermedium with a razor blade (Cook, 1965, Figure 2A). Nails were removed and the dorsal skin was pulled down towards the digits and removed. Hind paws were fixed in 4% paraformaldehyde (Thermo Fisher Scientific, Whitby, ON, Canada) for 5 to 6 days and demineralized in Cal-Ex II (Thermo Fisher Scientific, Whitby, ON, Canada) for 10 days. Tissues were then washed and soaked in deionized water for an hour to flush out the decalcifier. Hind paws were post-fixed in 4% paraformaldehyde for 6 h, dehydrated in graded ethanols, cleared in xylenes, and infiltrated with 1:1 IM/LP Histoplast paraffin mix (Thermo Fisher Scientific, Whitby, ON, Canada) in an automated tissue processor (HistoCore Pearl, Leica Biosystems Inc., Concord, ON, Canada). They were then embedded in the paraffin mix with the mid-sagittal plane of the hind paw parallel to the surface of the mold. The entire mouse foot was serially sectioned (RM2235, Leica Biosystems Inc., Concord, ON, Canada) at 8 μm in the sagittal plane, with four sections being floated onto each microscope slide (Superfrost Plus, Thermo Fisher Scientific, Whitby, ON, Canada). Each hind paw produced a series of approximately 130 slides for histological and immunohistochemical processing. To enable visualization and counting of Meissner-like corpuscles across the hind paw, every third slide obtained from each hind paw was stained using hematoxylin and eosin. This was conducted using an automated stainer (ST5010, Leica Biosystems Inc., Concord, ON, Canada) and coverslipping machine (CV5030, Leica Biosystems Inc., Concord, ON, Canada) to maximize reproducibility (Figure 2B).

### Immunohistochemistry

To confirm hematoxylin and eosin identification of Meissner-like corpuscles, two slides representing each group of interest (female and male, wild type and *itga1*-null) were selected for immunostaining of neurofilament-200 (NF200), a marker for myelinated sensory nerve fibers (Bakovic et al., 2018) and S100, a marker for Schwann cells (Miko and Gschmeissner, 1994). To visualize and count Merkel cells across the entire hind paw, every third slide of three female wild type and three female *itga1*-null hind paws (260 slides total) were immunostained for cytokeratin-20 (CK20), a specific marker for Merkel cells in mice (Moll et al., 1995). All slides processed for immunohistochemistry had two fully stained sections with antibodies, one omission control, and one reagent control.

Slides for NF200 were deparaffinized, quenched in 3% hydrogen peroxide for 30 min, and blocked with 5% normal goat serum for 2 h at room temperature (RT). Primary antibody against NF200 (N4142, 1:1,000, Sigma–Aldrich, St. Louis, MO, USA) was incubated overnight at 4°C. Secondary horseradish peroxidase-labeled anti-rabbit polymer (DAKO, Burlington, ON, Canada) was applied for an hour at RT the following morning. Slides for S100 and CK20 were deparaffinized and incubated with proteinase K (20 μg/ml in TE buffer, pH 8.0) at 40°C. Slides were then quenched in 3% hydrogen peroxide for 30 min and blocked with 5% normal goat serum (S100) or 3.6% normal horse serum (CK20) for 2 h. Primary antibody against S100 (ab34686, 2.5–5 μg/ml, Abcam Inc., Toronto, ON, Canada) or CK20 (MA1–35556, 1:100, Thermo Thermo Fisher Scientific, Rockford, IL, USA) was incubated overnight at 4°C. An ultra-sensitive avidin-biotin complex (ABC) peroxidase rabbit (S100) or mouse (CK20) IgG staining kit (Thermo Thermo Fisher Scientific, Rockford, IL, USA) was applied the following day. From the kit, the secondary biotinylated IgG was applied for an hour and the ABC was applied for 30 min at RT. All slides were developed using 3,3-diaminobenzidine (Sigma–Aldrich, Oakville, ON, USA) for 15 min, counterstained with Harris’ Hematoxylin (Thermo Fisher Scientific, Whitby, ON, Canada) for 2 s, dehydrated and cleared then mounted in Cytoseal^TM^ XYL (Thermo Thermo Fisher Scientific, Burlington, ON, Canada).

### Analysis

Meissner-like corpuscles and Merkel cells were identified by two independent blinded observers using light microscopy (Nikon Eclipse E400, Mississauga, ON, Canada). The mechanoreceptors were counted and their location across the hind paw mapped. Different regions of the hind paw were identified on each section including (from posterior to anterior) heel hair follicles, glabrous plantar metatarsal skin, glabrous footpad skin, ridged glabrous plantar skin/plantar hair follicles, glabrous plantar digit skin, and digit hair follicles (Figure 2C). To enable three different stains to be used, mechanoreceptors were counted on every third slide for each hind paw. To avoid double-counting of mechanoreceptors and to enable immunohistochemical control sections on each slide, Meissner-like corpuscles, and Merkel cells were counted on one section of every third slide. The number of Merkel cells on an entire slide was then extrapolated by multiplying by 2.5 [32/12.5; 32 μm being the total tissue thickness per slide (8 μm per section × 4 sections per slide) and 12.5 being the average diameter of a Merkel cell (10–15 μm)]. No such adjustment was required for the Meissner-like corpuscles whose diameter (10–30 μm) was similar to the total tissue thickness per slide (32 μm; Zelená, 1994). The total number of Meissner-like corpuscles and Merkel cells in each region of the hind paw was then determined by dividing the number of mechanoreceptors on each slide by the fraction of slides analyzed (1/3). To calculate mechanoreceptor density in the footpads, the total number of receptors per footpad was further divided by the surface area of the footpad, estimated as the surface area of a hemisphere (2 Πr^2^, where r is the radius). To calculate the radius of each footpad, the first and last slides where a footpad appeared were recorded, and the total number of slides (inclusive) containing the footpad was determined. This number was then multiplied by the tissue thickness on each slide (32 μm total) to obtain the diameter, and divided by two for the radius.

### Footfall Video Recording

Six wild type and six *itga1*-null adult mice (three of each sex) were placed on an elevated glass platform with a narrow tunnel for them to walk through. The mice were allowed to freely walk through the tunnel three times before a recording was taken. Videos (240 fps, 720 ppi) were taken on an iPhone 7 using the “SLO-MO” setting and with the flash on. The iPhone 7 autofocused on the glass plate and the plantar surface of the hind paw came into focus as it was in contact with the glass. The exceptional temporal and spatial resolution of the iPhone 7 enabled us to distinguish which parts of the hind paw (toes 1–5, footpads 1–6, heel) were touching the glass, in what order, and for how long. These data, similar to the human footfall sequence of the heel, mediolateral arch, and toe-off, cannot be collected using the more standardized Digigait system (often used to measure specific gait parameters such as stride length/width/frequency) due to its insufficient temporal and spatial resolution (150 fps, 179 ppi). For a good recording at least three footfalls of the right hind paw during continuous forward gait had to be clearly visible and in focus. The middle footfall of the right foot was then analyzed (Windows Media Player) and frames indicating a change in the contact area of the plantar hind paw surface were extracted.

### Statistics

Statistica (StatSoft Inc., Tulsa, OK) was used for all statistical analysis, with significance defined as *p* < 0.05. Footpad surface area and Meissner-like corpuscle and Merkel cell density in the footpads were analyzed using three-way repeated-measures ANOVA with independent categorical variables including footpad (1–6), sex (male, female), and genotype (wild type, *itga1-*null). Meissner-like corpuscle and Merkel cell count in the digits were analyzed using three-way repeated-measures ANOVA with independent categorical variables including digit (1–5), sex (male, female), and genotype (wild type, *itga1-*null). All values in the text are reported as mean ± SE.

## Results

### Mice

As expected, male mice of both genotypes were 7 g heavier than females (*p* < 0.01, males = 30.9 ± 1.3 g, females = 23.8 ± 1.4 g), and genotype did not affect mass (*p* = 0.39).

### Location of Mechanoreceptors

**Figure 3:**
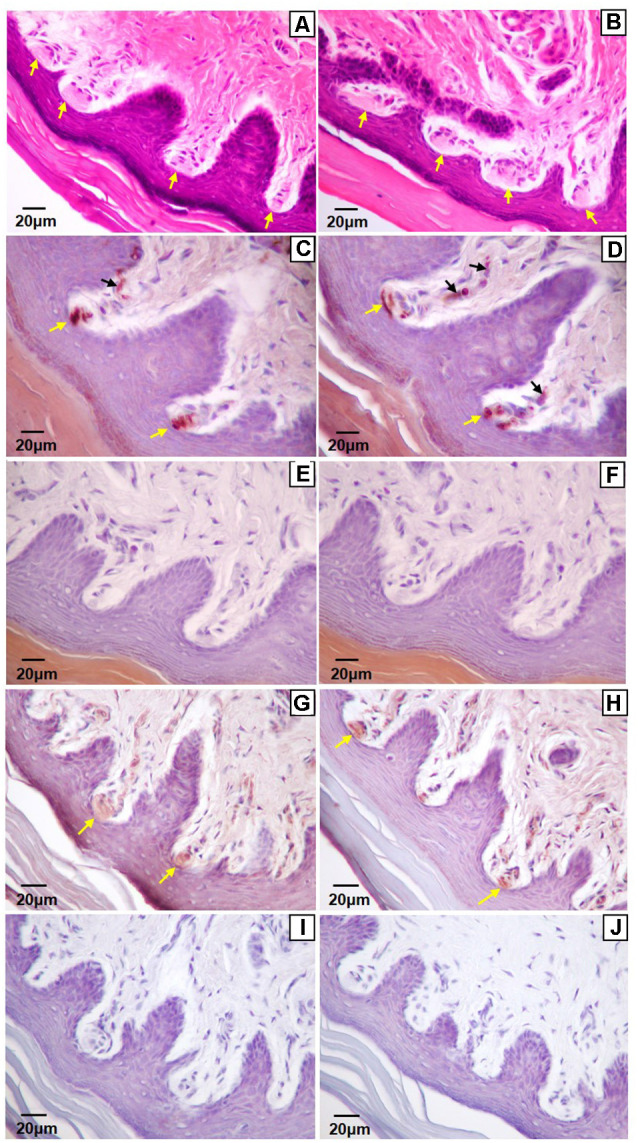
Light microscopy images of the glabrous footpad **(A,C–J)** and skin of digit tip **(B)** containing Meissner-like corpuscles (yellow arrows). Sagittal sections (8 μm) stained with hematoxylin and eosin **(A,B)**, or immunostained for neurofilament-200 **(C,D)** or S-100 **(G,H)** and counterstained with hematoxylin **(C–J)**. Controls for immunohistochemistry with secondary antibody only **(E,I)** and blank **(F,J)** are immediately below their respective stained images. Note the arising sensory neuron supplying the corpuscle from the dermis **(C,D**, black arrows).

**Figure 4:**
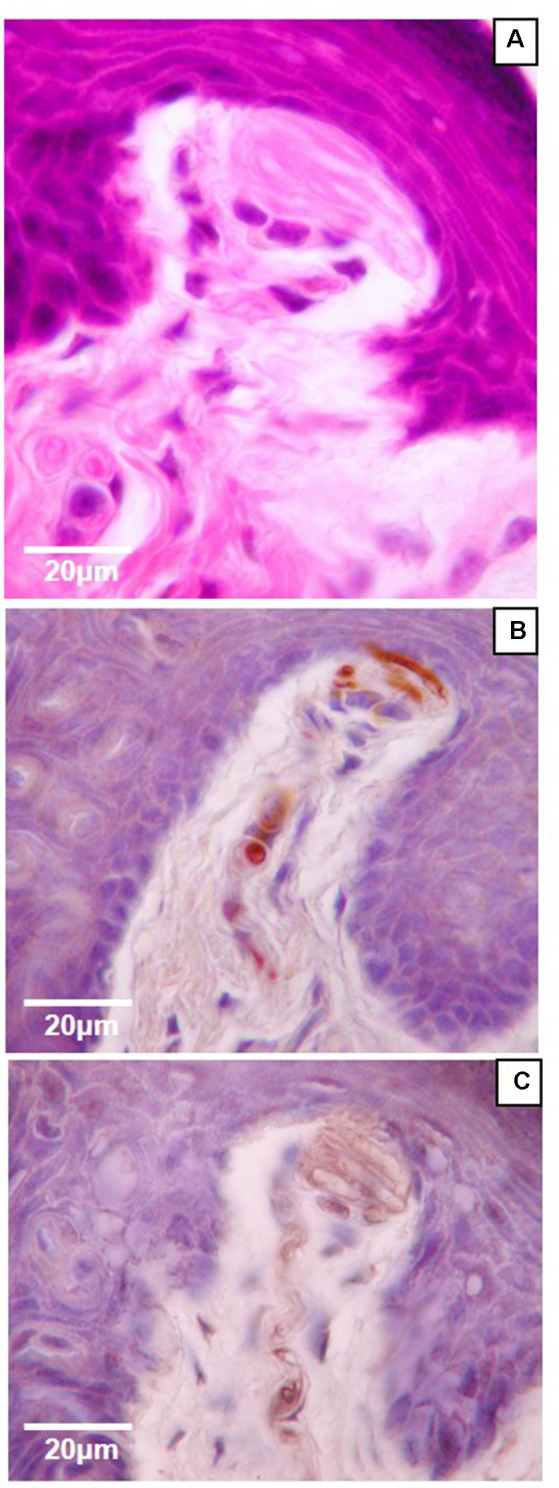
High magnification (100×) light microscopy images of Meissner-like corpuscles in the glabrous footpad. Sagittal sections (8 μm) stained with hematoxylin and eosin **(A)** or immunostained for neurofilament-200 **(B)** or S100 **(C)** and counterstained with hematoxylin **(B,C)**.

**Figure 5:**
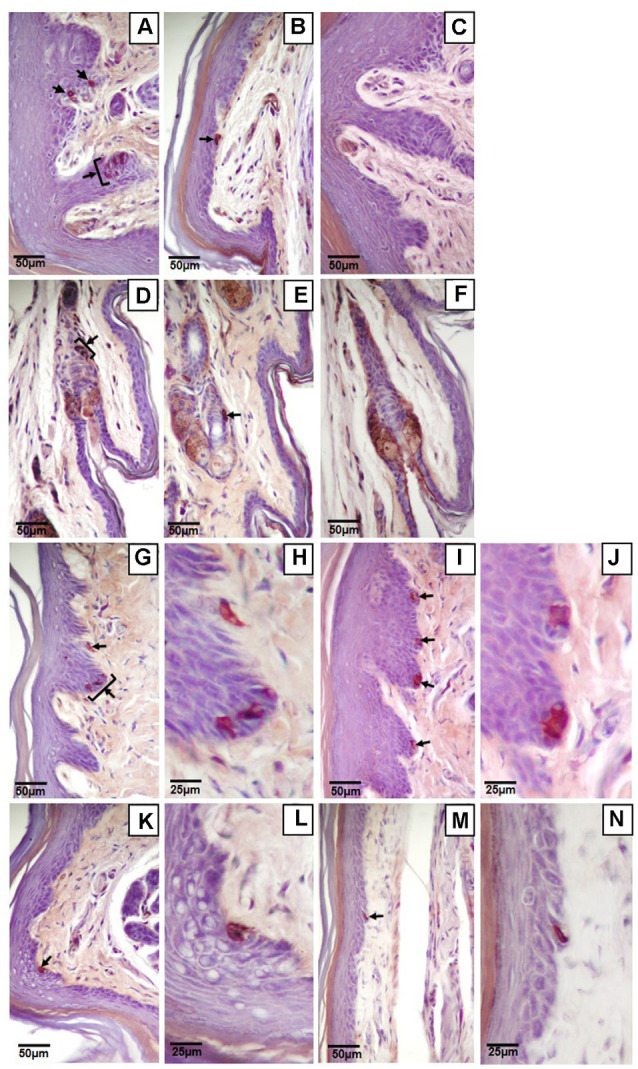
Light microscopy images of Merkel cells (black arrows) in the glabrous and hairy hind paw skin. Clustered **(A,D,G,H)** or single **(B,E,I–N)** Merkel cells in the glabrous plantar skin **(A,B)** or hair follicles **(D,E)** of the digit, the glabrous footpad skin **(G–J)**, the ridged glabrous plantar skin between footpads 2 and 3 **(K,L)**, and the glabrous plantar metatarsal skin **(M,N)**. Higher magnification images of Merkel cells **(H,J,L,N)** are on the immediate right of the corresponding lower magnification image **(G,I,K,M** respectively). Controls of the glabrous plantar skin **(C)** and hair follicles of the digit **(F)** with secondary antibody only. Sagittal sections (8 μm) immunostained for cytokeratin 20 and counterstained with hematoxylin.

Of the four mechanoreceptor types, only Meissner-like corpuscles and Merkel cells were found in hind paw skin. Pacinian corpuscles and Ruffini endings, typically identifiable with hematoxylin and eosin staining alone, were not observed despite close examination along the phalange, metatarsal, and tarsal bones of the hind paw. Hematoxylin and eosin staining revealed Meissner-like corpuscles exclusively in the dermal papillae of the glabrous footpad skin and the distal tip of the glabrous plantar digit skin (Figures 2B,C, 3A,B, 4A). They were distinguished by the light pink stained lamellar Schwann cells that appear cotton-like at the bulb of the dermal papillae. Their presence was confirmed using NF200 antibody to stain for sensory myelinated nerve fibers that supply and coil within the corpuscle (3C,D, 4B) and S100 antibody to stain for Schwann cells (3G,H, 4C). Myelinated nerve fibers were concentrated at the bulb of the dermal papillae intertwined with the cotton-like lamellar Schwann cells, with the remaining length of the nerve fiber supplying the corpuscle descending into the dermis. Merkel cells were not observed with hematoxylin and eosin staining alone, however immunostaining for CK20, which stains specifically for Merkel cells in mice (Moll et al., 1995), revealed these receptors in both the hair follicles and the glabrous skin (Figure 5). Specifically, Merkel cells were found (from posterior to anterior) in the heel hair follicles, glabrous plantar metatarsal skin, glabrous footpad skin, ridged glabrous plantar skin, plantar hair follicles, glabrous plantar digit skin, and digit hair follicles (Figures 2C, 5). In the skin, Merkel cells were found exclusively in the stratum basale of the epidermis. In areas where dermal papillae were present, Merkel cells were found only in the epidermal ridges (Figures 5A,G,I,K). In regions where hair follicles are present, Merkel cells were present in the bulge and outer root sheath of the hair follicle but not in the adjacent skin (Figures 5D,E). Non-specific, light brown staining was observed in the stratum corneum, muscle, sweat glands, and connective tissue for NF200, S100, and CK20 stained slides; however, dark brown staining of the antigens of interest was easily distinguishable from adjacent non-specific staining (Figures 3C–J, 5).

Genotype did not influence the total number of Merkel cells in the hind paw (wild type 2459, *itga1*-null 2210) and the vast majority of Merkel cells (78% wild type, 79% *itga1*-null) were seen in the digit hair follicles and the footpad glabrous skin (Table 1). Merkel cells were organized as single cells (Figures 5B,E,I–N) or clusters of two to six cells (a cell or two apart, Figures 5A,D,G,H), with most clustered Merkel cells (56%) presenting as two cell clusters (Table 1). In the wild type mouse, there were similar numbers of Merkel cells organized as single cells or clusters in all regions of the hind paw except the plantar metatarsal skin where there were more presenting alone than in clusters (Table 1). The number of single Merkel cells in all regions was similar in *itga1*-null compared to wild-type mice, however, there were fewer clustered Merkel cells in the digit hair follicles and more clustered Merkel cells in the footpad glabrous skin of *itga1*-null compared to wild type hind paws (Table 1).

**Table 1:** Number of single or clustered (2–6 cells) Merkel cells found in various regions of the hind paw in wild type or *itga1*-null mice.

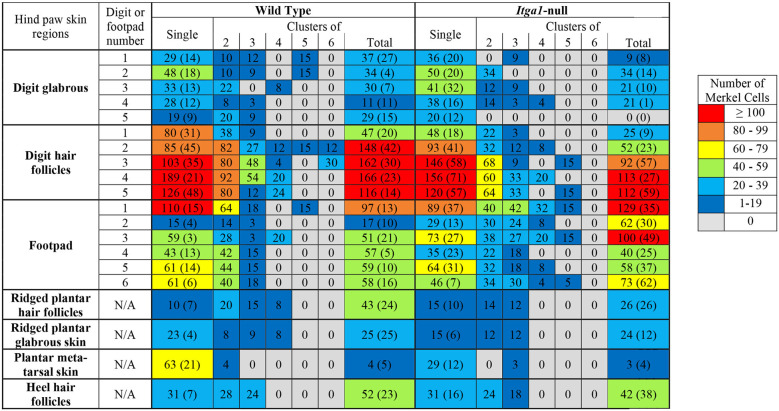

### Density of Mechanoreceptors in the Footpads

**Figure 6:**
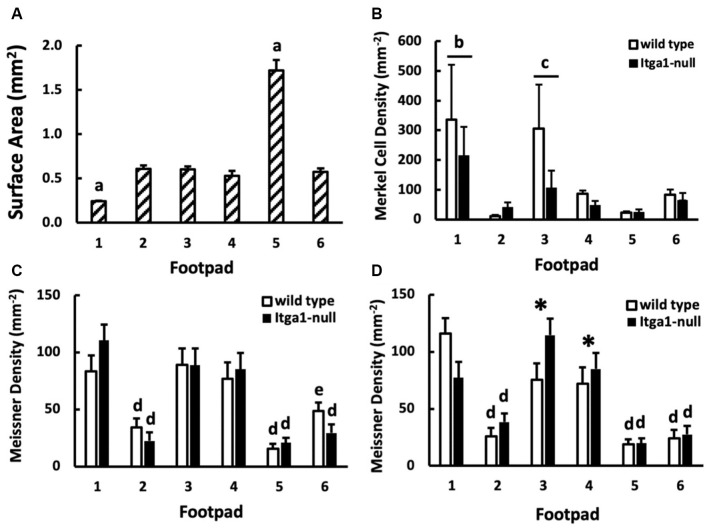
**(A)** Surface area (*n* = 16), **(B)** Merkel cell density (*n* = 3), and **(C,D)** Meissner-like corpuscle density across footpads 1–6 (*n* = 4). Merkel cell density is further compared between genotypes (wild type, *itga1*-null; **B)** and Meissner-like corpuscle density between genotypes and sexes [female **(C)**, male **(D)**]. Values are means with standard error bars. ^a^Different from all other footpads (*p* < 0.01). ^b^Different from all footpads except footpad 3. ^c^Different from footpad 2 and 5. ^d^Different from footpads 1, 3, 4 (*p* < 0.05). ^e^Different from footpads 1, 3, 4 (*p* < 0.05) except footpad 4 equivalent and different from footpad 5 equivalent. *Different from genotype equivalents (*p* < 0.01).

The location of the footpad influenced its surface area (*p* < 0.001, Figure 6A) however there was no effect of genotype or sex. The surface area of footpad 1 and footpad 5 was smaller and larger, respectively than all other footpads (*p* < 0.01). Footpads 2 ,3, 4, and 6 were similar in surface area (Figure 6A). Footpad location also influenced Merkel cell density (*p* = 0.01), however, genotype did not (Figure 6B). The density of Merkel cells in footpads 1 and 3 was, on average, more than eight times greater than footpads 2 and 5 (*p* < 0.05), indeed Merkel cell density in footpad one was at least three times that of footpads 2, 4, 5, and 6 (*p* < 0.01, Figure 6B). Meissner-like corpuscle and Merkel cell densities were comparable across footpads 2, 4, 5, and 6 (Figures 6B–D), however, in footpad 1 Merkel cell density was two to three times greater than Meissner-like corpuscle density. A three-way interaction between footpad number, sex, and genotype was evident for Meissner-like corpuscle density (*p* = 0.022). Meissner-like corpuscle density was larger in footpads one, three, and four compared to footpads 2, 5, and 6 (*p* < 0.05, Figures 6C,D). Interestingly, a genotype effect was observed in footpads 3 and 4 of male mice, where *itga1*-null mice had greater Meissner-like corpuscle density than their wild type counterparts (Figure 6D).

### Mechanoreceptor Count in the Digits

**Figure 7:**
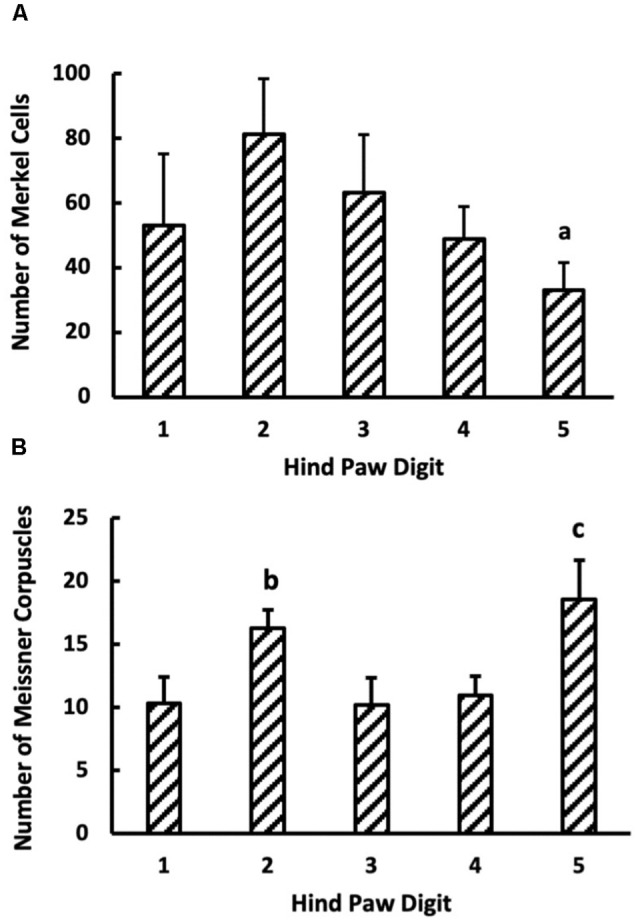
**(A)** Merkel cell (*n* = 6) and **(B)** Meissner-like corpuscles count in the glabrous skin of digits 1–5 (*n* = 16). Values are means with standard error bars. ^a^Different from digit 2 (*p* < 0.05). ^b^Different from digit 3 (*p* < 0.05). ^c^Different from digits 1, 3, 4 but not 2.

The number of Merkel cells in the digits was independent of digit location and genotype (Figure 7A). In contrast, digit location significantly influenced Meissner-like corpuscle number (*p* < 0.05), with the number of Meissner-like corpuscles in digit five being larger than digits one, three, and four (Figure 7B). Genotype and sex did not affect the number of Meissner-like corpuscles in the digits. Overall, the number of Merkel cells were on average 4.2 times greater than the number of Meissner-like corpuscles in the digits.

### Footfall Analysis

**Figure 8:**
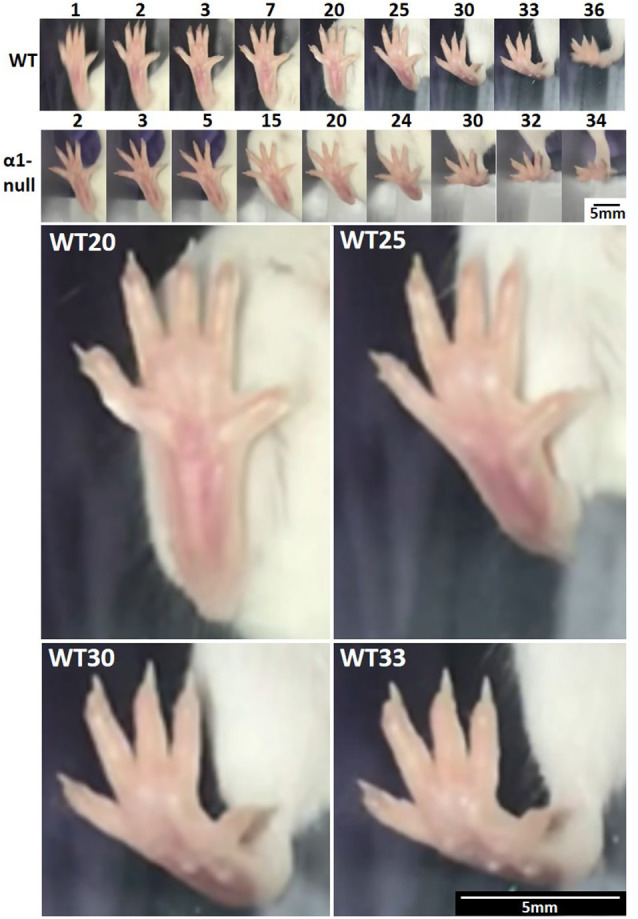
Mouse footfall analysis. Isolated frames from a right hind paw footfall during an uninterrupted stride of normal walking by a wild type (WT) and integrin α1-null (α1-null) female mouse (*n* = 12). Numbers above the images indicate frame numbers. Frames 20, 25, 30, and 33 from the footfall of the wild-type mouse have been enlarged below to demonstrate spatial resolution. Note how digits are splayed throughout footfall and that contact begins with digits 1, 2, and 5, followed quickly by the entire plantar surface. The heel and then the metatarsals are lifted, followed by digits 1 and 5, and finally digits 2, 3, and 4. Video captured at 240 fps, 720 ppi.

High-speed video capture for slow-motion analysis of uninterrupted normal walking revealed similar footfall patterns between wild type and *itga1*-null mice (Figure 8). For both the wild type and *itga1*-null mouse footfall lasted 120–150 ms and began with digits 1, 2, and 5 contacting the ground. Within 25 ms of this initial touch, the remaining digits (splayed mediolaterally) and the entire plantar surface of the foot of both wild type and *itga1*-null mice touched the ground. Liftoff for both mice began 40–60 ms later with the heel, then the metatarsals, then the phalanges of digits 1 and 5, and finally digits 2, 3 and 4 (Figure 8).

## Discussion

**Figure 9:**
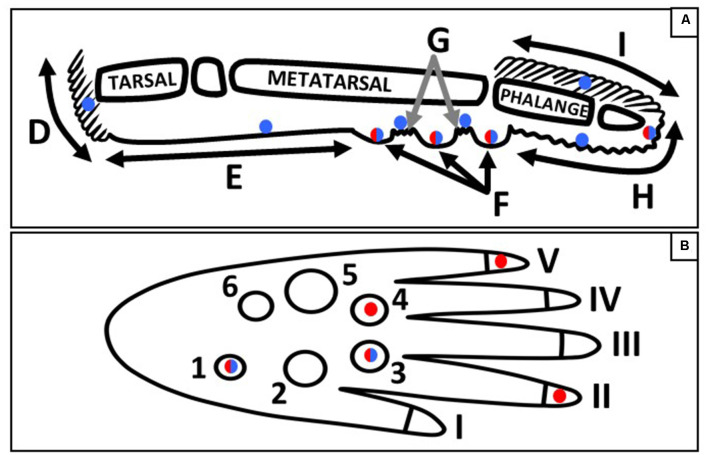
Schematic of the sagittal **(A)** and plantar **(B)** view of a mouse hind paw. Regions of the sagittal view are labeled; D, heel hair follicles; E, glabrous plantar metatarsal skin; F, glabrous footpad skin; G, ridged glabrous plantar skin/plantar hair follicles; H, glabrous plantar digit skin; and I, digit hair follicles; and the footpads and toes are numbered in the plantar view. Blue circles indicate Merkel cell locations, red circles indicate the footpads and toe tips with the largest densities of Meissner-like corpuscles, and red/blue circles indicate locations for both Meissner-like corpuscles and Merkel cells **(A)** or the footpads with the largest densities of both Meissner-like corpuscles and Merkel cells **(B)**.

The purpose of this study was to compare the distribution of mechanoreceptors across the hind paw skin of *itga1*-null and wild type mice. Also, footfall patterns during gait were analyzed as a first step in correlating mechanoreceptor distribution and functionality. Consistent with previous studies, Merkel cells and Meissner-like corpuscles were present in hind paw skin with Merkel cells present in all regions across both hairy and glabrous skin and Meissner-like corpuscles limited to glabrous footpads and digit tips (Figure 9A; Zelená, 1994; Albuerne et al., 2000; Fleming and Luo, 2013). Merkel cells were alone or in clusters of up to six cells, with the majority of clusters located in the glabrous footpad skin and digit hair follicles, whereas Meissner-like corpuscles were always alone. Moreover, our results reveal a greater number of Merkel cells than Meissner-like corpuscles in the digits (factor of 4) and footpad 1 (factor of 2–3). Pacinian corpuscles and Ruffini endings, typically identifiable with hematoxylin and eosin staining alone, were not observed despite close examination along the phalange, metatarsal, and tarsal bones of the hind paw. Previous histological investigations have confirmed the absence of Ruffini-like structures in the forepaw digit of the raccoon (Rice and Rasmusson, 2000) and have identified Pacinian corpuscles in forelimb phalanges, radius, ulna, fibula, tibia, and interosseous membranes of mice (Zelená, 1978; Sedý et al., 2004; Prsa et al., 2019), but not in the hind paw. Though, we cannot discount that our sampling approach [every third slide (88 μm)] may have missed these receptors that are few in number and ~50 μm in diameter (Prsa et al., 2019), it would make sense functionally that feedback regarding the high-frequency vibrations of phalanges in the forepaw may be critical for feeding and grooming, but not in the hind paw for sitting and gait (Hunt, 1961).

It is interesting to consider these data in parallel to measurements of single-unit neural recordings taken from mouse hind paws (Cain et al., 2001; Walcher et al., 2018). Neural recordings are reported from both RA and SA afferents, likely terminating in Meissner-like corpuscles or Merkel cells respectively, in both hairy and glabrous skin (Walcher et al., 2018) or in the glabrous skin of the plantar surface in addition to the toes and footpads (Cain et al., 2001). Furthermore, RA and SA afferent responses have been reported in equal numbers (Walcher et al., 2018) or with a larger proportion of RA (Meissner-like corpuscle, 71%) compared to SA (Merkel, 29%) responses (Cain et al., 2001). Also, some have divided the SA responses into equal proportions of SAI (Merkel) and SAII (Ruffini) responses (Walcher et al., 2018). These findings contradict our histological data that locates Meissner-like corpuscles only in the glabrous skin of the footpad or digit tip, a larger number of Merkel cells compared to Meissner-like corpuscles and no evidence of Ruffini endings. This mismatch between neural recordings and our histological findings may suggest that other sensory organs, not Meissner-like or Pacinian corpuscles, or Ruffini endings, may be in place across hind paw hairy skin and glabrous plantar metatarsal skin to transduce mechanical stimuli into the RA and SAII afferent responses recorded by electrophysiology, or that characterized mechanoreceptors can respond neurologically in novel ways (Wellnitz et al., 2010). Indeed, it has been proposed that SAII fibers may be innervating diffuse patterns of Merkel cells, but never Meissner-like or Pacinian corpuscles (Rice and Albrecht, 2008). Also, the neural recordings and our histological data support the hypothesis that multiple Merkel cells can be innervated by a single afferent nerve compared to Meissner-like corpuscles that may be innervated by multiple nerves. Indeed, 70% of Meissner-like corpuscles in mouse hind paw pad four are innervated by more than one nerve fiber (Walcher et al., 2018).

Merkel cells were most dense in footpads 1 and 3, and Meissner-like corpuscles most dense in footpads 1, 3, and 4, located in the posterior (footpad 1) and anterior (footpads 3 and 4) regions of the hind paw (Figure 9B). Across the digits, Merkel cells were evenly distributed however there were more Meissner-like corpuscles in digits 2 and 5 (located medially and laterally respectively) compared to the other digits (Figure 9B). Our footfall analysis revealed that mice initially contact the ground with the tips of the most medial and lateral digits (1, 2 and 5) followed rapidly by the entire plantar surface of the foot with the digits splayed. The mouse then lifts its foot starting from the posterior and moving to the most anterior regions. The higher concentration of Meissner-like corpuscles in digits 2 and 5 likely mediate balance during initial ground contact with the RAs associated with Meissner-like corpuscles known to provide sensory feedback regarding dynamic skin deformation and slip (Johnson, 2001). In contrast, the increased density of both Merkel cells and Meissner-like corpuscles in the most posterior footpad 1, and the most anterior footpads 3 and 4 may suggest their importance in sensing the change from sustained to dynamic skin deformation and thus coordinating removal of the posterior followed by the anterior regions of the foot from the ground. In particular, the high density of Merkel cells in the most posterior footpad suggests that they may be important in providing feedback of changes in center of pressure during the start of liftoff. Additionally, the mouse is constantly maneuvering between walking/running on all four limbs and sitting with the forepaws lifted. This could further explain the large density of mechanoreceptors in the most posterior footpad and a potential role in keeping the mouse from falling backward during sitting and mediating its sitting/walking transitions by providing feedback for the timing of muscle firing in the hindlimb (Zehr et al., 2014).

In agreement with our hypothesis, male *itga1*-null mice had a larger density of Meissner-like corpuscles in footpads 3 and 4 compared to wild type controls. It is important to note that the *itga1* deficit in the *itga1*-null mouse is present from conception, through birth, and as the pups mature and the skin, its afferents, and low-threshold mechanoreceptors develop (Feito et al., 2018). It is known that the interaction of dendrites with the epidermis influences dendrite branching and degeneration and that pruning of epidermal nerve endings can occur in response to interactions with keratinocytes (Takahashi et al., 2019; Yang and Chien, 2019). Similarly, we hypothesize that Meisner-like corpuscles are pruned in response to their interaction with keratinocytes in the epidermis and that mechanoreceptor density decreases less in the *itga1*-null mouse compared to controls due to disrupted mechanoreceptor-keratinocyte connection. We hypothesize that a similar process takes place to result in the spatially varied mechanoreceptor number and density patterns we report here across the digits, footpads, and plantar skin of the hind paw. That the gait experiences of the young pups as they develop normal function, and the resulting interactions between the mechanoreceptors and the epidermis, determines the spatial distribution of the mechanoreceptors across the plantar skin of the hind paw adult. It is known that the density of both Meissner’s corpuscles and Merkel cells in the distal phalanges of the hand decreases with age (20–90 years; García-Piqueras et al., 2019) and so it is perhaps even more conceivable that mechanoreceptor density can decrease in the early years during development. Future studies are warranted however to better understand the full extent of this adaptation both in terms of the magnitude of the decrease in mechanoreceptor density from birth to adulthood and the timeframe over which it occurs.

It is important to consider our findings within the limitations of the histological approach that we chose. Serially sectioning the hind-paw results in approximately 520 sections (130 slides). Due to our desire to perform three different staining techniques, to have control sections for immunohistochemistry, and to avoid double-counting, we counted mechanoreceptors on one section from every third slide (~88 μm). Consequently, our mechanoreceptor counts were extrapolated across the entire slide stack, which may not accurately represent sections that were not analyzed. Furthermore, we limited our manual immunohistochemical processing to the six female mice (three of each genotype) based on our preliminary data that showed genotypic effects and more mechanoreceptors in female compared to male mice, and thus our Merkel cell data may not be representative of male mice. In terms of Meissner-like corpuscles, however, the number of mechanoreceptors we calculate to be in footpad four (38.5 ± 2.2) is comparable to that counted by others using 3D confocal imaging (43.3 ± 3.4; Walcher et al., 2018).

In conclusion, we compared the distribution of mechanoreceptors across the hind paws of male and female *itga1*-null and wild type mice. Merkel cells and Meissner-like corpuscles were present, however, Ruffini endings and Pacinian corpuscles were not observed. Meissner-like corpuscles were located exclusively in the glabrous footpads and tip of the digit skin however Merkel cells were found in all regions of the hind paw in both hairy and glabrous skin. The increased density of Meissner-like corpuscles and Merkel cells in footpads 1, 3, and 4 suggests their role in contact timing (RAI) and in providing sensory information regarding pressure changes during liftoff and footfall (SAI). High concentrations of Meissner-like corpuscle in digits 2 and 5 support their role in signaling foot contact in the mediolateral direction and therefore influencing balance during footfall. Finally, a larger density of Meissner-like corpuscles in footpads 3 and 4 in male *itga1*-null mice compared to wild type controls paves the way for future single fiber *in vivo* recordings at the sciatic nerve and modality-specific stimulation in these footpads to provide important insight into the role of integrin α1β1 in tactile mechanotransduction.

## Data Availability Statement

The original contributions presented in the study are included in the article, further inquiries can be directed to the corresponding author.

## Ethics Statement

The animal study was reviewed and approved by the University of Guelph Animal Care Committee (AUP#3960).

## Author Contributions

VW, LB, and AC: study conception and design, data interpretation, and manuscript editing. VW, LR, and JM: data collection. VW, LR, and AC: data analysis. VW and AC: manuscript first draft. All authors contributed to the article and approved the submitted version.

## Conflict of Interest

The authors declare that the research was conducted in the absence of any commercial or financial relationships that could be construed as a potential conflict of interest.
